# The amino acid composition and protein quality of various egg, poultry meal by-products, and vegetable proteins used in the production of dog and cat diets

**DOI:** 10.3382/ps/pey462

**Published:** 2018-10-23

**Authors:** R A Donadelli, C G Aldrich, C K Jones, R S Beyer

**Affiliations:** 1 Grain Science & Industry Department, Kansas State University, Manhattan, KS 66506, United States of America; 2 Animal Sciences & Industry Department, Kansas State University, Manhattan, KS 66506-0100, United States of America

**Keywords:** pet food, poultry protein, vegetable protein, poultry by-products, protein efficiency ratio

## Abstract

New protein ingredients are used to support pet food market growth and the development of new products while maintaining animal dietary needs. However, novel protein sources (e.g., spray-dried chicken, and (or) rice, pea, and potato protein concentrates) have limited data available regarding their protein quality. The objective of this study was to evaluate protein ingredients used in the pet food industry by laboratory analysis and a chick growth assay as a model. Following analysis for proximate and amino acid composition, chicks (six birds per pen with four pens per treatment) were fed experimental diets for 10 d. Diets contained 10% crude protein from each of the experimental protein sources (spray-dried egg—SDEG; spray-dried egg white—SDEW, spray-dried inedible whole egg—SDIE, chicken by-product meal—CBPM, chicken meal—CKML, low-temperature fluid bed air-dried chicken—LTCK, low-temperature and pressure fluid bed dried chicken—LTPC, spray-dried chicken—SDCK, whey protein concentrate—WPCT, corn gluten meal—CGML, corn protein concentrate—CPCT, potato protein isolate—PPIS, rice protein concentrate—RPCT, pea protein isolate—PEPI, soy protein isolate—SPIS, and soybean meal—SBML) along with an N-free diet (negative control). Chicks fed SDEG, SDIE, and LTPC had the highest protein efficiency ratio (PER; *P* < 0.0001; 5.18, 5.37, and 5.33, respectively), LTCK and SDCK were intermediate (4.54 and 4.79), and the CBPM and CKML were the lowest among the poultry proteins for EAA:NEAA, PER, and Lys availability. Among the vegetable proteins, PPIS and SBML had the highest PER values (3.60 and 3.48, *P* < 0.0001). In general, the chick PER method ranked the quality of animal protein sources higher than vegetable proteins, and these results were consistent with the EAA:NEAA ratio and Lys availability.

## INTRODUCTION

The pet food industry in the US is a $23 billion trade with a growth rate of approximately 4% annually (Animal Protein Producers Industry, 2015). Dry pet foods (extruded or baked) constitute around 70% of the market with most processed via extrusion cooking (Pet Food Institute, 2012). Dry pet foods require a concentrated protein source to support the nutritional needs and product claims (e.g., high protein, low ash, high fat, grain-free, no by-product). These protein ingredients have traditionally been rendered animal by-products from the meat industry (Hertrampf and Piedad-Pascual, 2000). Among the animal proteins, poultry proteins are largely used in dry pet foods as well as wet foods (e.g., raw mechanically separated chicken).

To grow their business, pet food manufacturers must differentiate their product lines from competitors. Promoting their ingredient composition is a common strategy to distinguish and differentiate their products. Thus, creating demand for ingredients to formulate diets that spur consumer interest, i.e., meeting special dietary needs. Opportunities exist for the poultry industry to market new by-products via new recovery processing techniques developed to turn more secondary streams into valuable feed ingredients.

Some of these protein sources have been evaluated in an animal model (Johnson et al., 1998; Yamka et al., 2003, 2004; Dust et al., 2005). However, technology and material composition have changed over time. New data would benefit the pet and poultry industry. Proximate and amino acid composition and bioavailability data for single ingredients are helpful when making diet formulation decisions. Methods such as the cecectomized rooster (to assess digestibility, Johnson et al., 1998), and the chick protein efficiency ratio (**PER**; to rank different protein sources in regard to the amino acid composition, Cramer et al., 2007) have been used previously to evaluate single proteins. Meaningful information can be obtained from PER assays; however, like most in vivo methods, there are limitations to the technique, for example: moisture content of the ingredients, particle size differences in the ingredients, ingredients with high fat content, and ingredients with high mineral content can affect the results (Steinke, 1977). Evaluation with a biological model is very important when assessing new ingredients or new processing methods applied to existing ingredients. While chicks have different amino acid requirements to the dog or cat, they can still provide valuable information regarding the protein quality of ingredients due to their sensitivity to shortcomings in nutrients that might limit growth. Further, the PER technique allows the evaluation of a single ingredient that would not be permitted in a dog or cat model. It was our hypothesis that in combination with the proximate and amino acids composition data the chick PER model would provide rapid and meaningful ranking of various proteins for application in pet (cat and dog) food formulations.

### Objective

The objective of this study was to evaluate and compare the nutrient composition and protein quality of various egg, poultry, and vegetable proteins used in pet food manufacturing.

## MATERIALS AND METHODS

### Protein Sources and Feed Preparation

Dry powder ingredients including spray-dried egg (**SDEG**), spray-dried inedible whole egg (**SDIE**), low-temperature fluid bed air-dried chicken (**LTCK**), low-temperature and low-pressure fluid bed dried chicken (**LTPC)**, and spray-dried chicken (**SDCK**; American Dehydrated Foods, Inc., Springfield, MO), spray-dried egg white (**SDEW**; Oskaloosa Food Products Corp., Oskaloosa, IA), chicken by-product meal (**CBPM**), chicken meal (**CKML**), corn gluten meal (**CGML**), pea protein isolate (**PEPI**), soybean meal (**SBML**; Lortscher's Animal Nutrition, Inc., Bern, KS), whey protein concentrate (**WPCT**; Glanbia, Intl., Twin Falls, ID), corn protein concentrate (**CPCT**; Cargill Inc., Blair, NE), rice protein concentrate (**RPCT**; Beneo-Remy, Mannheim, Germany), potato protein isolate (**PPIS**; Roquette America, Keokuk, IA), and soy protein isolate (**SPIS**; ADM, Chicago, IL) were acquired prior to the study. Sub-samples were collected in triplicate (approximate 200 g each), mixed thoroughly, then an aliquot (approximate 200 g) was placed in whirl-pak bag for subsequent analysis. The ingredients were analyzed for proximate constituents (moisture, crude protein (**CP**), crude fat, crude fiber, and ash) at a University Laboratory (University of Missouri Agriculture Experiment Station chemical laboratory, Columbia MO) according to published AOAC standards (1980). Likewise, each ingredient amino acid composition (AOAC, 2012a), including tryptophan (AOAC, 2012b), was analyzed. After digestion with 6 N HCl for 24 h at 110°C (all amino acids except methionine, cysteine, and tryptophan), the amino acids were separated by ion-exchange chromatography and then the concentration was determined with a Beckman 6300 amino acid analyzer (Beckman, Palo Alto, CA). Methionine and cysteine were first oxidized by performic acid, respectively, to methionine sulfone and cysteic acid prior to acid hydrolysis. Tryptophan was hydrolyzed in 3 M mercaptoethanesulfonic acid before analysis. Available lys was determined by AOAC 975.44 method (AOAC, 2006) and lys availability (%) was calculated as the ratio of available lys to total lys.

A basal diet was formulated with all the nutrient requirements in accordance with the NRC (1994), except for protein and amino acid composition (Table 1). For each experimental diet, the protein source was added to the basal N-free ration to an inclusion that contributed 10% CP in replacement of the 2:1 cornstarch to dextrose mix (Johnson and Parson, 1997).

**Table 1.** Ingredient composition of the N-free diet.

Item	Percentage (as fed)
Ingredient	
Corn starch: dextrose (2:1, wt/wt)	to 100
Soybean oil	5.000
Mineral premix^1^	5.365
Vitamin premix^2^	0.203
Choline chloride	0.220
Tylan 40^3^	0.050

^1^Percentage of the diet: Ca_3_(PO_4_)_2_, 2.8; CaCO_3_, 0.3; CoSO_4_7H_2_O, 0.0001; CuSO_4_5H_2_O, 0.002; ferric citrate, 0.0415; H_3_BO_4_, 0.009; K_2_HPO_4_, 0.9; KI, 0.004; MgSO_4_7H2O, 0.35; MnSO_4_H_2_O, 0.065; Na_2_-MoO_4_2H_2_O, 0.0009; Na_2_SeO_3_, 0.00002; NaCl, 0.88; and ZnCO_3_, 0.01; total, 5.365.

^2^Supplied the following per kilogram of complete diet: vitamin A, 5,200 IU; vitamin D, 1,080 IU; vitamin E, 30 mg; vitamin B12, 0.04 mg; riboflavin, 10.0 mg; niacin, 50.0 mg; pantothenic acid, 27.6 mg; vitamin K, 2.0 mg; folic acid, 4.0 mg; vitamin B6, 5.0 mg; thiamin, 17.8 mg; and biotin, 0.6 mg.

^3^88.2 g/kg of Tylosin, Elanco Animal Health, Indianapolis, IN

### Chick and Experimental Design

The Kansas State University Institutional Animal Care and Use Committee approved the experiment and procedures. Chicks (408 1-d-old male broilers; Cobb × Cobb) were obtained from Cobb Vantress (Siloam Springs, AR). They were placed in Petersine battery cages and fed a common starter diet (23% CP corn–soybean meal starter diet) for 6 d to allow for acclimation to the experimental conditions. Fresh water in a trough at the end of each pen was available ad libitum throughout the duration of the experimental period. On d 7, fasted for 8 h prior to being chicks were weighed individually and allotted to treatment by weight into one of 68 pens (6 birds per pen) in a randomized complete block design, with battery representing block (n = 4). The chicks were housed in a temperature- (29°C) and humidity-controlled (65% R.H.) room with a 24 h light schedule. The birds were fed the experimental diets for 10 d, with feed available ad libitum in a trough feeder. At the end of the period, feed was removed and weighed. Birds were fasted overnight and then weighed by pen to determine weight gain.

Feed and chick weight were recorded to calculate PER and net protein ratio (**NPR**) according to equations 1 and 2:

(1) \begin{equation*} {\rm{PER = BWG}}/{\rm{CPI}} \end{equation*}

(2) \begin{equation*} {\rm{NPR = }}\left({{\rm{BWG}}-{\rm{GN}} - {\rm{free}}} \right)/{\rm{CPI}} \end{equation*}

wherein BWG is body weight gain in g, CPI is crude protein intake in g, and GN-free is the weight gain of chicks fed the N-free control diet in g.

Data were analyzed as a randomized complete block design using the GLIMMIX procedure of SAS (9.4). Means were separated by significant F (Steel and Torrie, 1984). Pen was considered the experimental unit with four replicates for each treatment and the battery (n = 4) was the block. Fisher's Protected LSD was used to control the pairwise comparisons of the treatments.

## RESULTS AND DISCUSSION

The performance of the chicks in this study were consistent with work from previous studies reported in the literature and conducted at this facility (Cramer et al., 2007). Most diets were readily consumed, environmental conditions remained stable, and the birds remained healthy throughout the course of the experiment.

The proximate analysis and most amino acid composition data from samples agrees with prior published research and standard reference tables (Morita and Kiriyame, 1993; Fialho et al., 1995; Murray et al., 1997; Johnson et al., 1998; Hertrampf and Piedad-Pascual, 2000; Schmidt et al., 2003; Yamka et al., 2003; Keegan et al., 2004; Norberg et al., 2004; Dust et al., 2005; Ayadin et al., 2008; Guimarães et al., 2008; Hou et al., 2008; AAFCO, 2013; Lammert et al., 2014; Agarwal et al., 2015). There are a few exceptions. As an example, SDIE was made by spray drying whole egg after removing 15% of the egg white. This deduction of egg white increased the fat content (37.32%) compared to the SDEG (34.75%, Table 2).

**Table 2.** Proximate analysis of protein manufactured co-products.

	Percentage				
Protein feedstuff	CP^1^	Moisture	Crude fat^1^	Crude fiber^1^	Ash^1^
Spray-dried egg, SDGE	49.95	7.98	34.75	0.10	5.65
Spray-dried egg white, SDEW	83.49	8.93	0.15	0.10	5.35
Spray-dried inedible whole egg, SDIE	49.02	6.55	37.32	0.08	4.17
Chicken by-product meal, CBPM	67.66	5.36	13.56	1.04	10.54
Chicken meal, CKML	66.89	4.09	12.68	1.93	15.33
Low temp fluid bed air dried chicken, LTCK	76.21	4.64	11.18	0.35	8.32
Low temp & pressure fluid bed dried chicken, LTPC	72.27	6.46	18.71	0.25	5.56
Spray-dried chicken, SDCK	45.17	3.12	48.62	0.07	4.33
Whey protein concentrate, WPCT	76.10	5.95	0.56	0.10	2.67
Corn gluten meal, CGML	62.03	10.94	1.26	1.16	1.32
Corn protein concentrate, CPCT	78.83	8.56	2.52	0.79	0.89
Potato protein isolate, PPIS	75.06	11.66	0.38	1.14	2.51
Rice protein concentrate, RPCT	68.88	8.06	1.00	0.54	1.22
Pea protein isolate. PEPI	78.73	8.02	4.67	0.14	4.63
Soy protein isolate, SPIS	89.08	5.04	0.66	0.10	4.01
Soybean meal, SBML	47.82	11.13	0.85	2.83	6.59

^1^Dry matter basis.

LTCK and LTPC are co-products from chicken fat and broth production. Once the fat and broth or stock have been pressed from the tissue, the resulting material resembles cooked ground meat and has a moisture content near 50 to 60%. This moisture is removed by drying at low pressure and temperatures until the product reaches a moisture of less than 10%, whereas the SDCK is whole mechanically separated chicken meat that was atomized in a hot air-drying chamber until it became a powder. The starting materials and processes differ for LTCK and LTPC, reflecting their nutrient composition (Table 2).

Potato protein isolate, RPCT, and PEPI were derived from a water-based process that solubilizes and removes starch then precipitates and isolates the proteins before being dehydrated (AAFCO, 2013; Kalman, 2014). This results in a concentrated protein powder that is very low in fat (Table 2). The PPIS in the current study had a lower CP content than samples evaluated by Lynch et al. (2012).

The amino acid composition of the ingredients on a dry matter basis on Table 3, and on a percentage of the total amino acid content is reported on Table 5. By experimental design all dietary treatments were mixed to include the same CP content from a single proteins source; therefore, the limiting amino acids of that particular test ingredient would limit chick growth. SDEG was used as a reference protein; therefore, its amino acid content (expressed as a percentage of the total amino acid content) was considered the standard used for comparing the amino acid content of the other test protein sources. SDEW, CGML, CPCT, and RPCT had lower lys compared to SDEG. Methionine was lower for CBPM, PPIS, PEPI, SPIS, and SBML compared to SDEG. Chicken meal and the corn proteins had lower trp contents. Phenylalanine was the limiting amino acid for LTCK and SDCK, val for LTPC, and arg for WPCT (Table 5).

**Table 3.** Amino acid composition (dry matter basis) of protein feedstuffs.

	g*kg^−1^																	
Protein feedstuff	arg^1^	cys	gly	his^1^	ile^1^	leu^1^	lys^1^	OHlys	met^1^	phe^1^	pro	OHpro	ser	tau	thr^1^	trp^1^	tyr	val^1^
Spray-dried egg, SDEG	3.27	1.14	1.91	1.29	2.93	4.67	4.14	0.08	1.74	3.01	1.90	0.01	3.14	0.07	2.37	0.87	2.27	3.65
Spray-dried egg white, SDEW	5.35	2.40	3.22	2.17	4.92	7.96	6.37	0.08	3.40	5.71	3.70	0.01	5.11	0.04	3.96	1.58	3.76	6.70
Spray-dried inedible whole egg, SDIE	3.15	1.10	0.02	1.25	2.91	4.58	4.02	0.06	1.70	2.92	1.81	0.01	2.93	0.05	2.27	0.85	2.13	3.56
Chicken by-product meal, CBPM	4.80	0.95	6.07	1.41	2.79	5.10	4.59	0.29	1.38	2.83	4.13	1.82	2.62	0.52	2.76	0.73	2.44	3.39
Chicken meal, CKML	4.48	0.63	6.54	1.30	2.56	4.59	4.36	0.38	1.35	2.54	4.17	2.30	2.11	0.36	2.38	0.56	2.22	3.03
Low temp fluid bed air dried chicken, LTCK	5.52	0.83	3.88	2.27	4.21	6.85	7.03	0.14	2.18	3.71	3.40	0.43	2.74	0.06	3.62	1.08	3.21	4.58
Low temp & pressure fluid bed dried chicken, LTPC	5.26	0.82	4.29	2.07	3.66	6.18	6.54	0.24	1.99	3.25	3.49	0.91	2.61	0.11	3.30	0.89	2.97	3.90
Spray-dried chicken, SDCK	3.01	0.47	2.76	1.34	2.12	3.54	3.86	0.15	1.14	1.87	1.95	0.59	1.49	0.17	1.86	0.65	1.97	2.25
Whey protein concentrate, WPCT	2.39	1.86	1.69	1.65	5.19	9.03	7.72	0.04	1.74	2.91	4.97	0.01	3.66	0.04	5.66	1.73	2.70	5.06
Corn gluten meal, CGML	2.18	1.16	1.88	1.31	3.01	11.77	1.18	0.06	1.70	4.50	6.40	0.00	2.89	0.08	2.18	0.47	3.63	3.19
Corn protein concentrate, CPCT	2.59	1.41	2.22	1.63	3.66	14.71	1.18	0.04	2.07	5.58	8.25	0.01	3.63	0.03	2.69	0.52	4.58	3.96
Potato protein isolate, PPIS	4.53	1.19	4.08	1.82	4.90	8.75	6.68	0.12	1.85	5.46	4.28	0.05	3.65	0.05	4.64	1.04	4.58	5.80
Rice protein concentrate, RPCT	5.95	1.47	3.11	1.70	3.33	6.25	2.55	0.02	1.93	4.13	3.38	0.00	3.26	0.09	2.55	1.03	3.90	4.67
Pea protein isolate, PEPI	7.06	0.85	3.42	2.09	4.20	7.20	6.39	0.04	0.90	4.81	3.63	0.00	3.58	0.04	3.13	0.93	3.35	4.50
Soy protein isolate, SPIS	6.92	1.05	3.78	2.33	4.58	7.38	5.73	0.02	1.18	4.93	4.78	0.00	3.59	0.03	3.20	1.47	3.51	4.80
Soybean meal, SBML	3.86	0.70	2.24	1.34	2.59	4.13	3.41	0.06	0.72	2.71	2.66	0.05	2.12	0.10	1.95	0.83	1.97	2.61

^1^Essential amino acids.

As for amino acids associated with structural or connective tissue proteins, CBPM and CKML meal had the highest contents for hydroxyproline (OHpro) and hydrodylysine (OHlys; Table 5). Additionally, SDCK had higher levels of OHlys.

Taurine was detected at measurable levels in some protein sources. It is not unprecedented that taurine was detected in some plant ingredients, Spitze et al. (2003) found some plant and fungi products with taurine levels similar to the values observed in this study.

The lys availability (Table 4) was relatively high (>90%) for most samples. However, CBPM, CKML, LTCK, CGML, and CPCT had lys availability less than 90%. The decrease in lys availability can occur due to higher temperatures during processing as a result of browning reactions (Hertrampf and Piedad-Pascual, 2000).

**Table 4.** Summary amino acid (AA) composition of protein feedstuffs, values on dry matter basis.

	Available	Lys	Total	Essential	Non-essential	
Protein feedstuff	lys, g*kg^−1^	Availability, %	AA, g*kg^−1^	AA^1^, g*kg^−1^	AA^2^, g*kg^−1^	EAA: NEAA
Spray-dried egg, SDEG	3.74	90.3	53.09	27.95	25.14	1.11
Spray-dried egg white, SDEW	6.12	96.0	92.58	48.13	44.45	1.08
Spray-dried inedible whole egg, SDIE	3.63	90.2	51.40	27.21	24.18	1.13
Chicken by-product meal, CBPM	3.86	84.1	67.47	29.78	37.69	0.79
Chicken meal, CKML	3.40	78.0	64.03	27.16	36.87	0.74
Low temp fluid bed air dried chicken, LTCK	6.29	89.6	78.32	41.03	37.29	1.10
Low temp & pressure fluid bed dried chicken, LTPC	6.26	95.8	73.91	37.04	36.87	1.00
Spray-dried chicken, SDCK	3.74	96.8	44.22	21.64	22.58	0.96
Whey protein concentrate, WPCT	7.35	95.2	84.06	43.08	40.98	1.05
Corn gluten meal, CGML	1.01	85.7	71.68	31.48	40.20	0.78
Corn protein concentrate, CPCT	1.05	88.9	88.42	38.59	49.83	0.77
Potato protein isolate, PPIS	6.37	95.4	87.02	45.46	41.56	1.09
Rice protein concentrate, RPCT	2.41	94.9	72.26	34.08	38.19	0.89
Pea protein isolate, PEPI	6.20	96.9	81.52	41.20	40.31	1.02
Soy protein isolate, SPIS	5.53	96.5	89.07	42.52	46.55	0.91
Soybean meal, SBML	3.18	93.4	51.30	24.15	27.15	0.89

^1^Essential amino acids: arg, his, ile, leu, lys, met, phe, thr, trp, and val.

^2^Non-essential amino acids: cys, gly, OHlys, pro, OHpro, ser, tau, tyr.

**Table 5.** Amino acid of protein feedstuffs as a percentage of total AA (dry matter basis).

	Percentage																	
Animal meal	arg^1^	cys	gly	his^1^	ile^1^	leu^1^	lys^1^	OHlys	met^1^	phe^1^	pro	OHpro	ser	tau	thr^1^	trp^1^	tyr	val^1^
Spray-dried egg, SDEG	6.2	2.1	3.6	2.4	5.5	8.8	7.8	0.1	3.3	5.7	3.6	0.0	5.9	0.1	4.5	1.6	4.3	6.9
Spray-dried egg white, SDEW	5.8	2.6	3.5	2.3	5.3	8.6	6.9	0.1	3.7	6.2	4.0	0.0	5.5	0.0	4.3	1.7	4.1	7.2
Spray-dried inedible whole egg, SDIE	6.1	2.1	3.7	2.4	5.7	8.9	7.8	0.1	3.3	5.7	3.5	0.0	5.7	0.1	4.4	1.6	4.1	6.9
Chicken by-product meal, CBPM	7.1	1.4	9.0	2.1	4.1	7.6	6.8	0.4	2.1	4.2	6.1	2.7	3.9	0.8	4.1	1.1	3.6	5.0
Chicken meal, CKML	7.0	1.0	10.2	2.0	4.0	7.2	6.8	0.6	2.1	4.0	6.5	3.6	3.3	0.6	3.7	0.9	3.5	4.7
Low temp fluid bed air dried chicken, LTCK	7.0	1.1	5.0	2.9	5.4	8.7	9.0	0.2	2.8	4.7	4.3	0.5	3.5	0.1	4.6	1.4	4.1	5.9
Low temp & pressure fluid bed dried chicken, LTPC	7.1	1.1	5.8	2.8	4.9	8.4	8.9	0.3	2.7	4.4	4.7	1.2	3.5	0.1	4.5	1.2	4.0	5.3
Spray-dried chicken, SDCK	6.8	1.1	6.2	3.0	4.8	8.0	8.7	0.4	2.6	4.2	4.4	1.3	3.4	0.4	4.2	1.5	4.5	5.1
Whey protein concentrate, WPCT	2.8	2.2	2.0	2.0	6.2	10.7	9.2	0.1	2.1	3.5	5.9	0.0	4.4	0.1	6.7	2.1	3.2	6.0
Corn gluten meal, CGML	3.0	1.6	2.6	1.8	4.2	16.4	1.6	0.1	2.4	6.3	8.9	0.0	4.0	0.1	3.0	0.7	5.1	4.4
Corn protein concentrate, CPCT	2.9	1.6	2.5	1.8	4.1	16.6	1.3	0.0	2.3	6.3	9.3	0.0	4.1	0.0	3.0	0.6	5.2	4.5
Potato protein isolate, PPIS	5.2	1.4	4.7	2.1	5.6	10.1	7.7	0.1	2.1	6.3	4.9	0.1	4.2	0.1	5.3	1.2	5.3	6.7
Rice protein concentrate, RPCT	8.2	2.0	4.3	2.3	4.6	8.7	3.5	0.0	2.7	5.7	4.7	0.0	4.5	0.1	3.5	1.4	5.4	6.5
Pea protein isolate, PEPI	8.7	1.0	4.2	2.6	5.1	8.8	7.8	0.1	1.1	5.9	4.5	0.0	4.4	0.1	3.8	1.1	4.1	5.5
Soy protein isolate, SPIS	7.8	1.2	4.2	2.6	5.1	8.3	6.4	0.0	1.3	5.5	5.4	0.0	4.0	0.0	3.6	1.7	3.9	5.4
Soybean meal, SBML	7.5	1.4	4.4	2.6	5.0	8.1	6.6	0.1	1.4	5.3	5.2	0.1	4.1	0.2	3.8	1.6	3.8	5.1

^1^Essential amino acids.

Chick body weight gain was greatest (*P* < 0.05) for birds fed the SDEG, SDIE, and LTPC (185.8, 178.7, and 177.0 g, respectively, Table 6). The other animal-based proteins, SDEW, CBPM, LTCK, and SDCK had the second highest weight gain (108.5, 105.8, 147.3, and 150.2 g, respectively). The poorest gain in the poultry based proteins was recorded for the CKML treatment (78.5 g). The poorest (*P* < 0.05) weight gains were reported for the WPCT, CGML, and CPCT in which birds lost weight and performed similar to those fed the N-free basal diet (−7.5, −1.75, and −7.6 vs −18.6 g, respectively). This may in large part be due to the lower feed intake driven by the AA profile (Picard et al., 1993). The lower chick performance from corn-derived protein sources were mostly a function of AA profile when compared with SDEG. Cramer (2003) reported similar results even after adding 0.2% lys to CGML containing diets fed to chicks in a similar experimental design. The chicks fed the PPIS and SBML diets gained a similar amount of weight, though much less than those chicks fed the SDEG (87.1 and 93.5 vs 185.8 g). Chicks fed SPIS had lower weight gain (56.3 g) than PPIS and SBML. Finally, RPCT and PEPI had even lower weigh gain (29.0 and 32.3 g, respectively).

**Table 6.** Growth performance, protein efficiency ratio (PER), and net protein ratio (NPR) of chicks fed various protein feedstuffs (n = 4).

Treatment	BW Gain, g/chick	Feed Intake, g/chick	G:F	CP intake, mg/chick	PER	NPR	PER % of Spray-dried Egg
1. N-free basal diet	−18.6^j^	119.2^h^	−0.163^j^	–	–	–	–
2. As 1 + 10% CP from spray-dried egg	185.8^a^	359.0^a^	0.520^a,b^	35.9^a^	5.18^a^	5.70^a^	100.0^a^
3. As 1 + 10% CP from spray-dried egg white	108.5^c^	263.5^d,e^	0.410^d^	26.3^d,e^	4.12^c^	4.82^c^	79.4^c^
4. As 1 + 10% CP from spray-dried inedible whole egg	178.7^a^	332.4^b^	0.538^a^	33.2^b^	5.37^a^	5.93^a^	103.7^a^
5. As 1 + 10% CP from chicken by-product meal	105.8^c,d^	294.4^c^	0.360^e^	29.4^c^	3.59^d^	4.22^d^	69.3^d^
6. As 1 + 10% CP from chicken meal	78.5^f^	270.1^d^	0.288^f^	27.0^d^	2.91^e^	3.60^e^	56.1^e^
7. As 1 + 10% CP from low temp fluid bed air dried chicken	147.3^b^	324.3^b^	0.455^c^	32.4^b^	4.54^b^	5.12^b^	87.6^b^
8. As 1 + 10% CP from low temp & pressure fluid bed dried chicken	177.0^a^	329.0^b^	0.533^a^	32.9^b^	5.33^a^	5.90^a^	102.9^a^
9. As 1 + 10% CP from spray-dried chicken	150.2^b^	313.8^b,c^	0.480^b,c^	31.4^b,c^	4.79^b^	5.38^b^	92.4^b^
10. As 1 + 10% CP from whey protein concentrate	−7.5^i,j^	87.2^i^	−0.090^i^	8.7^h^	−0.90^h^	1.25^h^	−17.3^h^
11. As 1 + 10% CP from corn gluten meal	−1.75^i^	106.5^h,i^	−0.020^h^	10.7^h^	−0.19^g^	1.57^g^	−3.7^g^
12. As 1 + 10% CP from corn protein concentrate	−7.6^i,j^	96.6^h,i^	−0.080^i^	9.7^h^	−0.80^h^	1.13^h^	−15.4^h^
13. As 1 + 10% CP from potato protein isolate	87.1^e,f^	242.6^e^	0.360^e^	24.3^e^	3.60^d^	4.38^d^	69.5^d^
14. As 1 + 10% CP from rice protein concentrate	29.0^h^	157.7^g^	0.180^g^	15.8^g^	1.81^f^	3.00^f^	34.9^f^
15. As 1 + 10% CP from pea protein isolate	32.3^h^	168.8^g^	0.190^g^	16.9^g^	1.90^f^	3.01^f^	36.7^f^
16. As 1 + 10% CP from soy protein isolate	56.3^g^	194.1^f^	0.290^f^	19.4^f^	2.89^e^	3.85^e^	55.7^e^
17. As 1 + 10% CP from soybean meal	93.5^d,e^	269.0^d^	0.348^e^	26.9^d^	3.48^d^	4.17^d^	67.1^d^
Pooled SEM	4.85	8.08	0.0147	0.82	0.136	0.109	2.61
*P* =	<0.0001	<0.0001	<0.0001	<0.0001	<0.0001	<0.0001	<0.0001

^a–j^Within a column, means without a common superscript differ *P <* 0.05.

Feed intake for each treatment was ranked somewhat similar to chick weight gain, wherein the chicks ate more of the diet containing SDEG (359 g) than all the others. The chicks fed the SDIE, LTCK, LTPC, and SDCK had a slightly lower (*P* < 0.05) intake (332.4, 324.3, 329.0, 313.8 g, respectively) for all other treatments the chick feed intake was below 300 g. As expected, the G: F and the CP intake data have similar trends in statistical significances as gain and intake accordingly.

The PER accounts for the chick weight gain per unit of protein intake and was highest for the chicks fed the SDEG, SDIE, and LTPC (5.18, 5.37, and 5.33 g*g^−1^, respectively). The PER for chicks fed the LTCK and SDCK were lower (*P* < 0.05; 4.54 and 4.79 g*g^−1^, respectively), followed by those fed SDEW (4.12). The PER for CBPM was similar to SBML, and greater (*P* < 0.05) than CKML and SPIS (*P* < 0.05; 3.59 and 3.48 vs 2.91 and 2.89 g*g^−1^, respectively). The PER of RPCT and PEPI were 1.81 and 1.90 g*g^−1^, respectively, and WPCT, CGML, and CPCT were each less than zero indicating that chicks lost weight even when consuming the diet containing these ingredients. The NPR accounts for protein cost for maintenance and was determined by gain (or loss) of the chicks fed the N-free diet (Cramer et al., 2007). Given that the same loss was subtracted from all treatments, the relationship among all treatments was similar to that of the PER and the absolute values were greater by the same proportion amongst all dietary treatments.

The chick PER assay is an effective tool for determining the quality of a single ingredient as the chicks were fed an otherwise nutritionally adequate diet with the same quantity of protein (10% CP in each experimental diet, except the N-free diet). Therefore, growth was limited by the availability of the limiting amino acids alone. The assay was valuable for differentiating ingredients based on raw material composition (e.g., meat vs connective tissue) and processing techniques (e.g., low vs high drying temperature) that can affect nutrient availability and animal performance. Within this study the rank-order and (or) differences between the treatments remained the same whether evaluating the PER or the PER % of egg. However, using the SDEG as a reference allows for comparison of the results from this study to other studies in which SDEG was fed, and it creates an easy basis for understanding the relative rank of a protein to other contemporary proteins used in animal diets. Further, SDEG is a near-perfect protein for the chick and commonly used as a “standard” reference for these types of experiments (Johnson and Parson, 1997).

In summary, among the egg proteins, the chicks fed SDEW had a lower PER % of SDEG, which may be due to a lower proportion of lys compared to the SDEG and SDIE. Birds fed CBPM and CKML performance may have been lower due to decreased lys availability (reflecting some degree of heat damage), and due to significant amounts of connective tissue from collagen and elastin found in the joints and bone residue used to produce this ingredient (as evidenced by the lower EAA:NEAA and elevated OHlys and OHpro values). This agrees with the results reported by Johnson and Parson (1997) and Johnson et al., (1998), wherein they found lower PER and decreased digestibility in cecectomized rooster for these types of poultry proteins. Among the low-temperature fluid bed dried chicken proteins, the chicks fed the LTCK had lower performance, which was most probably due to lower proportions of met, phe, trp, and val. For the birds fed the LTPC, their performance was similar to those fed SDEG, which would suggest that this was chicken meat without connective tissue that was processed with minimal heat. Among the chicken meat proteins, the chicks fed the SDCK had the lowest PER, which is most likely a result of starting material composition as reflected by the lower EAA:NEAA, and the lower proportions of ile, met, phe, and val. Whey protein concentrate is often used as a protein for body building and nutritional support in parenteral diets for humans because it has a high protein digestibility-corrected amino acid score (Hoffman and Falvo, 2004). However, in this study it appears that WPCT had a significant departure from SDEG in its provision of limiting amino acids for support of chick growth. In a nutritionally complete diet, the performance of chicks fed whey decreased slightly, but unlike our study the intake was not affected (Szczurek et al., 2013; Malik et al., 2015). Whether the dramatic reduction in intake was a function of this experimental model or negative feedback due to the amino acid imbalance (WPCT had lower proportions of arg, his, met, phe, and val relative to SDEG) for the chick is not known.

Vegetable proteins are often deficient in at least one limiting amino acid. This can be exaggerated by thermal processing during their production. In this study, chicks fed corn-derived proteins (CGML and CPCT) performed as expected based on work by Cramer (2003), most probably due to the low proportion of lys, and in this case available lys. Chicks fed the potato protein (PPIS) also performed poorly, due to smaller feed consumption and lower met content when compared to SDEG. Tusnio et al. (2011) reported lower amino acid availability for potato protein concentrate when fed to young pigs, which could be a contributing factor to the decreased PER value reported here. Like the other vegetable protein concentrates, chicks fed RPCT and PEPI performed poorer than birds fed SDEG, most probably due to the limitation in available lys for RPCT and met for PEPI (Tomoskozi et al., 2001; Hou et al., 2008). Both of the soy proteins had limitations in essential AA proportions, especially met, which is commonly understood (Norberg et al., 2004). Thus, while the novel vegetable proteins like PPIS, RPCT, and PEPI are finding their way into pet food, they do have some limitations that must be accounted for in pet food formulas. Understanding these differences among these protein sources and which ingredients to pair with them to compensate for their deficiencies should lead to sufficient performance in a complete diet. However, limited ingredient or single ingredient pet foods are becoming more popular, and pose challenges when trying to balance the amino acid content, especially because of the amino acid limitations of some ingredients.

In conclusion, the chick PER assay was an effective method to evaluate a number of proteins new to the pet food industry and to rank single ingredients simultaneously in order to better understand their limitations based on the AA profile. In general, animal-based proteins had a more complete amino acid profile than the plant-based proteins, and this was reflected in better chick performance. The egg proteins had the highest PER values, followed by the LTCK. Chicken meal and CBPM were much lower in PER and may have been influenced by the content of amino acids from structural proteins (OHlys and OHpro) and reduction of essential amino acids from processing damage. The vegetable proteins all had limiting amino acids that severely reduced chick growth and PER vales, and would require amino acid supplementation or complementary proteins to become sufficient for complete dietary adequacy.
